# Maternal high-fat diet during lactation impairs thermogenic function of brown adipose tissue in offspring mice

**DOI:** 10.1038/srep34345

**Published:** 2016-09-30

**Authors:** Xingwei Liang, Qiyuan Yang, Lupei Zhang, Joseph W Maricelli, Buel D. Rodgers, Mei-Jun Zhu, Min Du

**Affiliations:** 1 Department of Animal Sciences, Washington State University, Pullman, WA 99164, USA; 2 State Key Laboratory for Conservation and Utilization of Subtropical Agro-bioresources, Guangxi High Education Laboratory for Animal Reproduction and Biotechnology, Guangxi University, Nanning, Guangxi 530004, China; 3 Institute of Animal Science, Chinese Academy of Agricultural Sciences, Beijing 100193, China; 4 School of Food Science, Washington State University, Pullman, WA 99164, USA

## Abstract

Maternal obesity and high-fat diet (HFD) predisposes offspring to obesity and metabolic diseases. Due to uncoupling, brown adipose tissue (BAT) dissipates energy via heat generation, mitigating obesity and diabetes. The lactation stage is a manageable period for improving the health of offspring of obese mothers, but the impact of maternal HFD during lactation on offspring BAT function is unknown. To determine, female mice were fed either a control or HFD during lactation. At weaning, HFD offspring gained more body weight and had greater body fat mass compared to the control, and these differences maintained into adulthood, which correlated with glucose intolerance and insulin resistance in HFD offspring. Adaptive thermogenesis of BAT was impaired in HFD offspring at weaning. In adulthood, HFD offspring BAT had lower *Ucp1* expression and thermogenic activity. Mechanistically, maternal HFD feeding during lactation elevated peripheral serotonin, which decreased the sensitivity of BAT to sympathetic β3-adrenergic signaling. Importantly, early postnatal metformin administration decreased serotonin concentration and ameliorated the impairment of offspring BAT due to maternal HFD. Our data suggest that attenuation of BAT thermogenic function may be a key mechanism linking maternal HFD during lactation to persisted metabolic disorder in the offspring.

There is concrete evidence supporting the association between maternal obesity and high energy intake with obesity and metabolic dysfunction in offspring^1^,^2^. Feeding the dam with high-fat diet (HFD) is the most common method to induce maternal obesity and over-nutrition in rodents^3^,^4^,^5^,^6^. Previous studies have demonstrated that lactation HFD predisposes offspring to obesity and metabolic disorder in rats^5^, and severely affects offspring metabolic profiles and neural development^4^. However, the mechanistic link between lactation diets and offspring metabolic dysfunction remains to be established.

There are two different adipose tissues, namely white adipose tissue (WAT) and brown adipose tissue (BAT)^7^,^8^,^9^, which have distinct characteristics and functions. The function of WAT is to store energy while BAT dissipates energy as heat^10^. Due to its high efficiency in oxidizing glucose and fatty acids and the recent discovery of its abundancy in adult human^11^,^12^,^13^,^14^, BAT is a critical organ against obesity and diabetes^15^,^16^,^17^. Consistently, activation of BAT by cold-exposure increased whole-body energy expenditure, glucose homeostasis and insulin sensitivity in humans^18^,^19^. Thermogenic activity of BAT can be activated or inhibited via the alteration of many factors including hormones and dietary bioactive compounds^20^,^21^,^22^. Serotonin (5-hydroxytryptamine, 5-HT)^23^ is a hormone elevated due to diet-induced obesity (DIO)^24^,^25^. Recently, ablation of serotonin genetically or pharmacologically was shown to prevent HFD-induced obesity through activation of BAT thermogenic function^25^,^26^, suggesting a possible mediatory role of serotonin in BAT function.

The fetal and neonatal stages are critical for BAT development, which is expected to have long-term impacts on offspring BAT function^27^. The objectives of the current study is to determine the impact of maternal HFD during lactation on the thermogenic function of offspring mice. We found that maternal HFD during lactation permanently attenuated BAT thermogenesis in offspring, which was partially mediated by enhanced serotonin signaling. Intervention of dams with metformin during lactation could protect offspring BAT function from impairment by maternal HFD.

## Results

### Maternal high-fat diet during lactation affected body composition and blood profiles of offspring at weaning and adulthood

**Figure 1:**
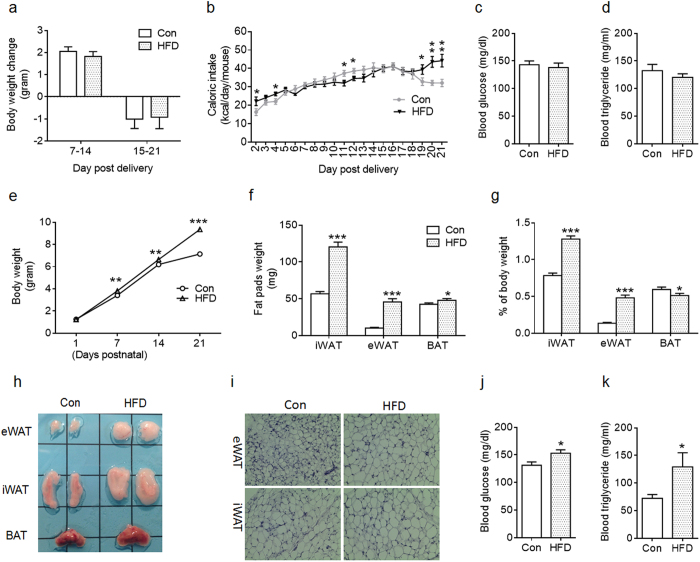
Characteristics of dams fed with Control (Con) and high-fat diet (HFD) during lactation and their pups at weaning. (**a,b**) Body weight change (**a**) and caloric intake (**b**) of Con and HFD dams were monitored during lactation (n = 9 Con and n = 8 HFD). (**c,d**) Blood glucose (**c**) and triglyceride levels (**d**) of Con and HFD dams at weaning (n = 6 per group). (**e**) Offspring body weight were monitored each week during sucking (n = 42 per group). (**f–h**) Fat tissue weight (BAT: brown adipose tissue; iWAT: inguinal adipose tissue; eWAT: epididymal adipose tissue) (**f**), ratio of fat tissue to body weight (**g**) and image of fat pads at weaning (**h**) (n = 11 Con and n = 12 HFD). (**i**) Representative images of eWAT and iWAT sections stained with H&E. (**j,k**) Blood glucose (**j**) and triglyceride levels (**k**) of Con and HFD offspring at weaning (n = 8 per group). Data are presented as Means ± S.E.M. *P < 0.05; **P < 0.01; ***P < 0.001 as determined using a Student’s *t*-test.

The dams were fed with Con or HFD throughout lactation, and no difference in body weight was observed (Fig. 1a). Con dams tended to have lower caloric intake during the beginning and the end of nursing period (Fig. 1b). There was no difference in blood glucose and triglyceride levels between Con and HFD dams at weaning (Fig. 1c,d). Milk triglyceride (Con vs HFD; 364 ± 37.3 vs 528 ± 67.1 mg/ml; *P* < 0.05) and insulin (Con vs HFD; 5 ± 0.9 vs 10 ± 1.5 ng/ml; *P* < 0.01) levels were elevated in HFD dams at weaning.

The offspring of HFD dams gained more body weight at postnatal day 7 (PND7) compared to Con (Fig. 1e), and such difference maintained throughout lactation (Fig. 1e). At weaning, HFD offspring had greater inguinal white adipose tissue (iWAT) and epididymal white adipose tissue (eWAT) mass, and higher ratios of fat weight to the body weight compared to Con offspring (Fig. 1f–h). Maternal HFD offspring also had greater BAT mass (Fig. 1f,h), but a significantly lower ratio of BAT weight to the body weight compared to Con (Fig. 1g). Consistent with the greater iWAT and eWAT mass, white adipocytes appeared to undergo hypertrophy in HFD offspring (Fig. 1i). Higher blood glucose and triglyceride levels were detected in HFD offspring (Fig. 1j,k). Taken together, maternal HFD feeding during lactation accelerated body weight gain and changed blood profiles of offspring at weaning.

**Figure 2:**
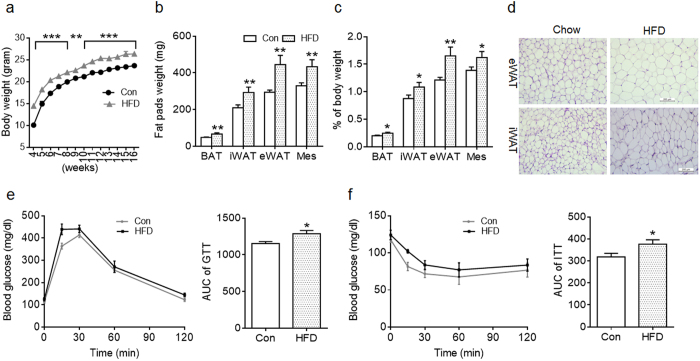
Maternal high-fat diet feeding during lactation reprograms body composition and metabolic syndrome in adult offspring. (**a**) Body weight of offspring from control (Con) and high-fat diet (HFD) feeding dams (n = 8 per group). (**b,c**) Fat tissue weight (BAT: brown adipose tissue; iWAT: inguinal adipose tissue; eWAT: epididymal adipose tissue; Mes: mesenteric fat) (**b**) and ratio of fat tissue to body weight (**c**) in adult offspring at 16-week of age (n = 8 Con and n = 6 HFD); (**d**) Representative images of eWAT and iWAT sections stained with H&E. (**e,f**) Glucose tolerance test (GTT; at 14-week of age) (**e**) and insulin tolerance test (ITT; at 15-week of age)) (**f**) of adult offspring (n = 5 per group). AUC: area under curve. Data are presented as Means ± S.E.M. *P < 0.05; **P < 0.01; ***P < 0.001 as determined using a Student’s *t*-test.

To analyze the long-term effect, offspring of Con and HFD dams were weaned on a chow diet up to 16 weeks of age when mice were euthanized. Offspring of HFD dams continued to have greater body weight compared to Con (Fig. 2a). Consistently, fat pad weight, and the ratios of individual fat weight to the body weight of HFD offspring were also higher (Fig. 2b,c), indicating adiposity in HFD offspring in adulthood. Adipocyte sizes of iWAT and eWAT of HFD offspring appeared to be larger than those of Con (Fig. 2d). Consistent with adiposity and large adipocytes, glucose tolerance and insulin sensitivity were impaired in HFD adult offspring (Fig. 2e,f). Together, maternal HFD during lactation has lasting effects on adiposity and metabolic disorders in offspring.

### Maternal HFD feeding during lactation affected *Ucp1* expression in BAT of offspring at weaning

**Figure 3:**
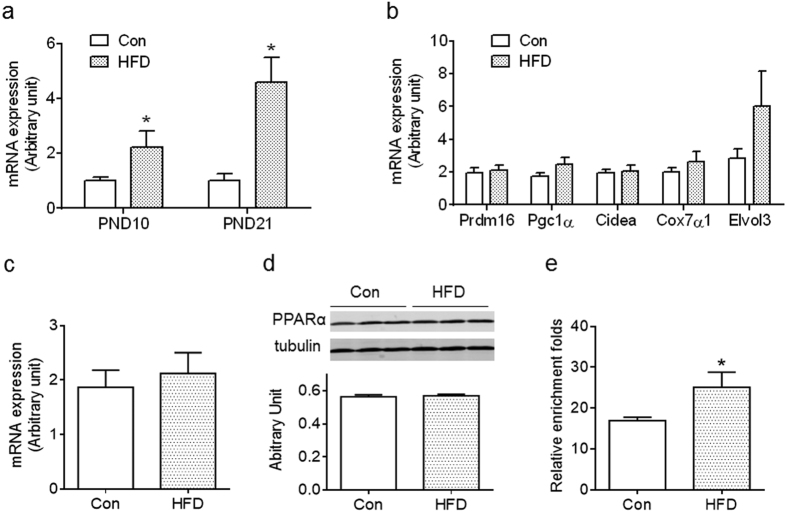
Maternal high-fat diet feeding during lactation elevates BAT *Ucp1* expression in offspring preweaning. (**a**) *Ucp1* expression in offspring BAT of control (Con) and high-fat diet (HFD) dams at PND10 and PND21 (n = 4–6 per group). (**b**) mRNA expression of BAT-selective genes in BAT of Con and HFD offspring at weaning (n = 6 per group). (**c,d**) PPARα mRNA (**c**) and protein contents (**d**) in BAT of Con and HFD offspring at weaning (n = 6 per group). (**e**) Binding of PPARα to the *Ucp1* promoter was elevated in HFD BAT as assayed by ChIP-PCR (n = 4 per group). Data are presented as Means ± S.E.M. *P < 0.05 as determined using a Student’s *t*-test.

To examine the impact of maternal HFD on *Ucp1* expression, BAT was collected at PND10 and PND21. Unexpectedly, offspring of HFD dams had higher *Ucp1* expression than Con (Fig. 3a). Of interest, maternal HFD had no effect on the expression of other brown fat marker genes, *Prdm16, Pgc1α, Cidea, Cox7α* and *Elvol3* (Fig. 3b). Because PPARα activates *Ucp1* expression in BAT^28^,^29^, we analyzed mRNA and protein contents of PPARα and found no difference between Con and HFD offspring (Fig. 3c,d). We further examined PPARα binding to the *Ucp1* promotor using ChIP-PCR and found that more PPARα bound to the *Ucp1* promoter of HFD BAT compared to Con (Fig. 3e). Thus, maternal HFD feeding during lactation induced *Ucp1* expression in BAT of offspring at weaning, likely mediated by recruiting PPARα to the *Ucp1* promoter.

### Maternal HFD feeding during lactation impaired thermogenic activity of offspring BAT at weaning and adulthood

**Figure 4:**
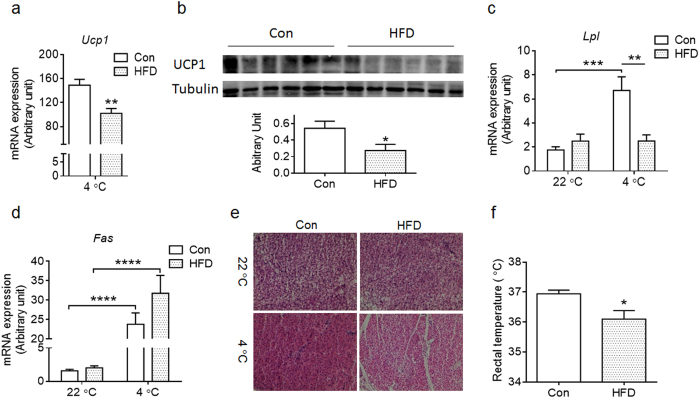
Maternal high-fat diet feeding during lactation impairs BAT thermogenic capacity of offspring at weaning under cold stimulus. Control (Con) and high fat diet (HFD) offspring at PND21 were subjected to a cold stimulus for 6 h at 4 °C. (**a,b**) *Ucp1* mRNA (**a**) and protein (**b**) levels in offspring BAT after cold exposure. (**c,d**) *Lpl* (c) and *Fas* (**d**) expression in BAT of offspring at room temperature (22 °C) and after cold exposure (4 °C) (n = 6 per group). (**e**) Histological changes of interscapular BAT from offspring of Con and HFD in room temperature and under cold exposure. (**f**) Rectal temperature of Con and HFD offspring at weaning after cold exposure (4 °C for 6 h) (n = 7 Con and n = 6 HFD). Data are presented as Means ± S.E.M. *P < 0.05; **P < 0.01; ***P < 0.001; ****P < 0.0001 as determined using a Student’s *t*-test or, where appropriate, two-way ANOVA and Tukey’ multiple comparison.

Given the unexpectedly higher *Ucp1* expression in HFD BAT at weaning, we further examined the response of *Ucp1* expression to cold exposure. Though the BAT of HFD offspring had higher *Ucp1* expression at room temperature (Fig. 3a), the *Ucp1* mRNA and protein contents were not elevated by cold exposure, in contrast to the Con BAT, where cold exposure dramatically increase *Ucp1* expression (Fig. 4a,b). These data show the impairment of thermogenic adaption of HFD offspring to cold stimulus.

Lipoprotein lipase (LPL) is activated by β-adrenergic signaling, which releases free fatty acids for BAT absorption and oxidation, while fatty acid synthase (FAS) catalyzes fatty acid synthesis. Both of these enzymes regulate the availability of free fatty acids, which provide fuels for thermogenesis in BAT^30^. We found that *Lpl* expression in BAT was induced by cold exposure in Con offspring, but not in HFD BAT (Fig. 4c). *Fas* expression in BAT was similarly induced both in Con and HFD BAT by cold exposure (Fig. 4d). In addition, no obvious difference in histological structures was found between Con and HFD BAT at room temperature (Fig. 4e), but under cold stimulus, Con BAT became denser, with less fat droplets compared to that of room temperature, suggesting effective oxidation of fatty acids for thermogenesis in Con BAT, in contrast to the HFD BAT where no obvious reduction in lipid droplets was detected (Fig. 4e)^3^. Accordingly, HFD offspring had lower rectal temperature under cold exposure when compared to Con offspring (Fig. 4f). Because the major physiological function of BAT is to maintain body thermostasis, the lower body temperature of HFD offspring under cold exposure confirmed the impairment in BAT thermogenic function. All these results demonstrated that the adaptive thermogenesis in BAT was impaired in HFD offspring at weaning.

**Figure 5:**
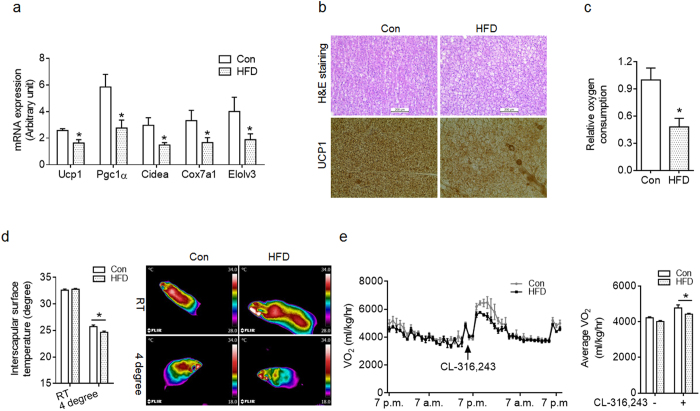
Maternal high-fat diet has long-term negative effects on BAT function of adult offspring at 4 months of age. (**a**) mRNA expression of genes enriched in the BAT of control (Con) and high-fat diet (HFD) adult offspring (n = 7 each group). (**b**) Representative images of offspring BAT of Con and HFD feeding dams stained with H&E (top) and immunohistochemically with UCP1 antibody (bottom). (**c**) Oxygen consumption of isolated BAT from adult offspring under room temperature (n = 4 per group). (**d**) Dorsal interscapular surface temperature of Con and HFD offspring under room temperature and 4 °C for 6 h (n = 4–5 each group). Right (**d**), representative thermal images of mice under room temperature and 4 °C. (**e**) Oxygen consumption of Con and HFD adult offspring in basal status and injected with CL316,243 (n = 4 per group). Data are presented as Means ± S.E.M. *P < 0.05 as determined using a Student’s *t*-test or, where appropriate, two-way ANOVA and Tukey’ multiple comparison.

Next, we examined the long-term effect of maternal HFD on thermogenic function in offspring BAT. Samples were collected from mice at 16-week of age. We then determined the mRNA expression of brown fat-selected genes. The HFD BAT had lower *Ucp1, Pgc1α, Cidea, Cox7α1* and *Elolv3* expression compared to Con (Fig. 5a). There was noticeable histological difference in BAT between HFD and Con offspring (Fig. 5b). The HFD BAT had bigger brown adipocytes with larger fat droplets (Fig. 5b), which was in agreement with higher BAT mass in HFD adult offspring (Fig. 2b,c). Furthermore, less UCP1 was detected in HFD compared to Con BAT (Fig. 5b). To determine BAT function, interscapular BAT was isolated from mice under room temperature and immediately analyzed oxygen consumption *ex vivo*. Minced HFD BAT had less oxygen consumption compared to Con indicating impairment of BAT function (Fig. 5c). BAT function was further examined by measurement of interscapular surface temperature. There was no difference in interscapular surface temperature in room temperature, but HFD offspring was lower than Con offspring under cold-stimulus (Fig. 5d). HFD offspring had a slight reduction in basal oxygen consumption compared to Con offspring (Fig. 5e). When injected with CL-316, 243, a β3-specific agonist targeting BAT, the oxygen consumption was higher in Con than in HFD offspring (Fig. 5e), suggesting that lactation HFD impaired BAT function. There was no difference in daily food intake between Con and HFD offspring (Con: HFD; 3.4 ± 0.11 versus 3.6 ± 0.14 g; n = 8), suggesting that the adiposity and metabolic dysfunctions in HFD offspring was not due to change in caloric intake. Taken together, maternal HFD feeding during lactation attenuated BAT thermogenesis in adult offspring.

### Maternal HFD during lactation affected BAT thermogenesis through inhibition of β3-adrenergic signaling

**Figure 6:**
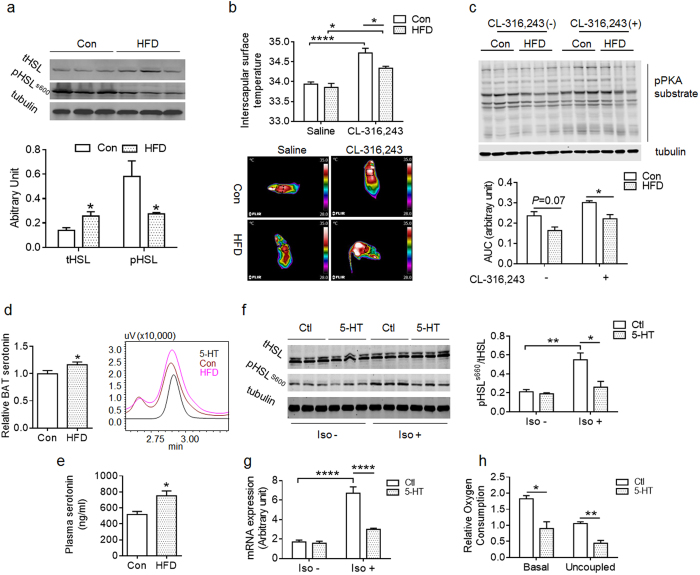
Inhibition of β3-adrenergic signaling in BAT of HFD offspring due to elevated serotonin. (**a**) Total and phosphorylated HSL (S660) in BAT of Con and HFD offspring at one-month of age (n = 6 per group, 3 shown). (**b**) Dorsal interscapular surface temperature of one-month old Con and HFD offspring at 20 min following an injection with saline or CL-316,243 (n = 4 per group; bottom: representative thermal images). The circle indicated that the area used for dorsal interscapular surface temperature measurement. (**c**) Protein kinase A (PKA) substrate phosphorylation in BAT of Con and HFD offspring at one-month age at 20 min following an injection with saline or CL-316,243 (n = 4 per group; upper: representative western blot). (**d**) Relative serotonin level in interscapular BAT of Con and HFD offspring at weaning (Normalized to Con; n = 4 per group; right (**d**), representative serotonin level determined by HPLC). (**e**) Serotonin level in plasma of adult offspring of Con and HFD dam (n = 5 per group). (**f**) HSL (S600) phosphorylation in control and isoproterenol-stimulated (Iso) brown adipocytes (n = 3 per group; (**f**) left, representative western blot). (**g**) *Ucp1* expression in control and isoproterenol-stimulated (Iso) brown adipocytes pretreated with serotonin. (**h**) Basal and uncoupled respiration rate of brown adipocytes pretreated with vehicle or serotonin as performed in (**f**). Serotonin  =  5-HT (5-hydroxytryptamine). *P < 0.05; **P < 0.01 and ****P < 0.0001 as determined using a Student’s *t*-test or, where appropriate, two-way ANOVA and Tukey’ multiple comparison.

To understand the underlying molecular mechanisms contributing to the impairment of BAT activity in HFD offspring, β3-adrenergic signaling pathway was examined in offspring at one-month of age. As a key effector of β3-adrenergic signaling, hormone-sensitive lipase (HSL) activity is essential for fatty acid oxidation in BAT. Total HSL was higher, while HSL phosphorylation was lower in BAT HFD offspring compared to Con, indicating the inhibition of β3-adrenergic signaling in the BAT of HFD offspring (Fig. 6a). There was no difference in basal interscapular surface temperature between Con and HFD mice (Fig. 6b), while the temperature of Con mice was higher than that of HFD offspring under CL-316, 243-stimulation (Fig. 6b). In agreement, the phosphorylation of basal PKA substrates was slightly lower in BAT of HFD compared to Con (*P* = 0.07), while the HFD had significantly lower PKA substrate phosphorylation under the treatment by β3-adrenoceptor agonist CL-316, 243 (Fig. 6c). These data indicated that the HFD BAT was less sensitive to β3-adrenergic agonist stimulation.

Next, we explored the underlying mechanisms leading to inhibition of β3-adrenergic signaling in HFD BAT. Because peripheral serotonin inhibits thermogenesis in BAT^26^, we measured serotonin contents in offspring. The HFD BAT had higher serotonin content at weaning (Fig. 6d) and also higher circulatory serotonin than that of Con in adulthood (Fig. 6e). To test whether elevated serotonin inhibited β3-adrenergic signaling in HFD BAT, we pretreated brown adipocytes with serotonin *in vitro*, and found that serotonin had no effect on basal HSL phosphorylation (Fig. 6f). But under the stimulation of isoproterenol, a β-adrenergic receptor agonist, serotonin inhibited HSL phosphorylation (Fig. 6f). Accordingly, there was no difference of basal *Ucp1* expression in brown adipocytes with/without serotonin treatment, but serotonin inhibited *Ucp1* expression under the stimulation of isoproterenol (Fig. 6g). The basal and uncoupling cellular respiration of brown adipocyte was reduced due to serotonin treatment (Fig. 6h). These data showed that elevated peripheral serotonin inhibited β3-adrenergic signaling in BAT of maternal HFD offspring.

### Metformin intervention improved BAT thermogenic activity in HFD offspring

**Figure 7:**
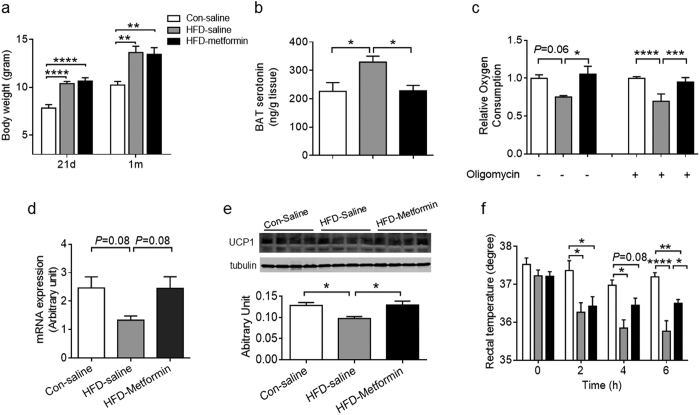
Early postnatal metformin treatment ameliorates thermogenic impairment in BAT due to maternal HFD feeding during lactation. (**a**) Body weight of Con-saline, HFD-saline and HFD-metformin at PND21 and one-month of age (Con-saline: Con offspring injected with saline; HFD-saline: HFD offspring injected with saline; HFD-metformin: HFD offspring injected with metformin from PND2-PND21, then weaned on chow diet at PND21. n = 7–11 each group). (**b**) BAT serotonin content of Con-saline, HFD-saline and HFD-metformin mice at one month of age (n = 6 each group). (**c**) Coupled and uncoupled respiration rate of isolated BAT from Con-saline, HFD-saline and HFD-metformin mice at one-month of age (n = 4 per group). (**d,e**) UCP1 mRNA (**d**) and protein contents (**e**) in BAT of Con-saline, HFD-saline and HFD-metformin at one-month of age under cold-stimulus for 6 h (n = 6 per group). (**f**) Rectal temperature changes of Con-saline, HFD-saline and HFD-metformin mice at one-month of age during 6 h under cold exposure (n = 6–8 per group). Data are presented as Means ± S.E.M. *P < 0.05; **P < 0.01; ***P < 0.001 and ***P < 0.0001 were determined by One-way ANOVA with Tukey’s post doc test.

Metformin is the first-line drug for treating diabetes. To study whether early postnatal intervention could protect the offspring BAT from the impairment due to maternal HFD feeding, the offspring of Con and HFD were administered with saline or metformin during lactation. Metformin treatment did not affect the body weight gain resulting from maternal HFD feeding (Fig. 7a). However, metformin inhibited the elevation of serotonin in HFD BAT (Fig. 7b). Importantly, metformin enhanced basal and uncoupled oxygen consumption of BAT, showing the improvement of BAT thermogenic activity (Fig. 7c). Although metformin treatment had no effect on *Ucp1* mRNA expression in BAT (HFD-saline versus HFD-metformin, *P* = 0.08) (Fig. 7d), the UCP1 protein was higher in HFD-metformin compared to HFD-saline under cold-stimulation (Fig. 7e). There was no difference in the rectal temperature among three groups at the beginning of cold exposure (0 h). But at the end of cold exposure (6 h), rectal temperature of HFD-saline was lower than that of Con-Saline mice, while HFD-metformin mice had higher rectal temperature compared to HFD-saline (Fig. 7f). Taken together, early postnatal metformin intervention protected the BAT function of HFD offspring.

## Discussion

Previous studies showed that neonates at the lactation stage is highly sensitive to reprogramming effects of maternal diets in rodents^4^,^5^. In this study, we examined whether and how the maternal HFD feeding during lactation affects thermogenic activity in BAT of mice offspring. We found that lactation HFD impaired BAT thermogenic function of offspring which was associated with persistent adiposity and metabolic syndrome. We further demonstrated that the impairment of BAT thermogenesis was partially due to the attenuation of cellular β3-adregernic signaling, which was linked to the elevation of peripheral serotonin by maternal HFD. More importantly, early postnatal metformin intervention ameliorated the impairment of BAT function in HFD offspring.

In this study, we observed that HFD offspring at weaning and in adults had excessive body weight gain, due to higher fat accumulation. Furthermore, HFD offspring developed glucose intolerance and insulin insensitivity in adulthood. However, there were no differences in body weight and blood profiles between Con and HFD dams during lactation, suggesting that the metabolic dysfunction in HFD offspring was not due to maternal metabolic disorder, but a specific alteration of maternal diet composition and then the changes of milk compositions^4^. This is quite different from maternal DIO model, where dams were fed HFD up to several weeks before mating and throughout the whole gestation and lactation stages, which led to maternal adiposity, alterations of blood profiles and development of glucose intolerance in dams^31^,^32^,^33^,^34^. In our study, HFD offspring continued to have greater body weight associated with reduction in oxygen consumption and unaffected caloric intake, suggesting that the adiposity of HFD adult offspring was primarily derived from reduced energy expenditure, likely associated with impaired BAT function.

To test whether impairment of BAT function in HFD offspring is one of the primary reasons leading to reduced energy expenditure, we first examined the *Ucp1* expression in BAT. Surprisingly, *Ucp1* expression was elevated in HFD BAT at PND10 and weaning, with no changes in the expression of other BAT markers. Despite higher *Ucp1* expression, the transcription levels of other BAT marker gene *Prdm16, Pgc1α, Cidea, Cox7a1* and *Elovl3* were unchanged in BAT of HFD offspring at weaning, suggesting there were defects in BAT functions. It was further confirmed by the fact that adaptive thermogenesis was attenuated in HFD BAT. The elevation of *Ucp1* expression was consistent with an earlier report where postnatal over-nutrition due to small litter size increases BAT *Ucp1* expression in rats^3^. HFD offspring gained more weight at weaning, which could be due to excessive energy intake from milk^4^ and also attenuated BAT adaptive thermogenesis in HFD offspring.

PPARα is a critical transcription factor regulating *Ucp1* expression^35^, and we found that there was more PPARα bound to the *Ucp1* promoter, providing an explanation for the elevated *Ucp1* expression in HFD BAT at weaning. HFD feeding induces the *Ucp1* expression in brown adipose tissue in rodents^36^. The higher fatty acids in milk of HFD dam increases fatty acid supply to pre-weaning offspring^4^, which might activate PPARs to increase *Ucp1* expression^28^,^37^.

The thermogenic function of BAT is tightly regulated by cellular β-adrenoceptor signaling pathway^14^. Cold stimulus activates BAT thermogenesis via the β3-adrenergic signaling, which involves PKA activation and HSL phosphorylation^14^,^38^. To further examine BAT function, we subjected offspring at weaning to cold temperature^20^,^39^, which stimulated *Ucp1* expression in Con BAT, but absent in HFD BAT, showing the cold-stimulated β3-adrenergic signaling was impaired in HFD BAT. Consistently, *Lpl* expression also was lower in HFD offspring than Con, whereas *Lpl* is essential for triglyceride uptake into BAT under cold condition^40^. Furthermore, there was obvious histological difference between room temperature and cold-stimulated BAT of Con offspring, which were absent in BAT of HFD offspring, reinforcing the notion of attenuated cold-stimulated thermogenic signaling in HFD BAT. In agreement with above data, at weaning, the rectal temperature of HFD offspring was lower compared to Con offspring under cold-stimulation. In adult, HFD BAT had lower expression of BAT marker genes, *Ucp1, Pgc1α, Cidea, Cox7a1* and *Elovl3* compared to Con BAT. Consistently, BAT of HFD offspring had reduced oxygen consumption and impaired thermogenic capacity under cold stimulus. Thus the attenuation of BAT function is a linking mediator of the long-term effect of maternal HFD on adiposity and metabolic disorder in offspring.

Serotonin is separately secreted by the central nervous system and peripheral tissue^41^, which regulates whole-body energy balance^42^,^43^. Obesity is known to elevate peripheral serotonin level^24^,^25^,^26^. Both genetically and pharmacologically knockdown peripheral serotonin protects the mice from HFD induced obesity via elevating β3-adrenergic pathway and BAT thermogenesis^26^. We found that maternal HFD offspring had higher peripheral serotonin level than Con at weaning and in adulthood. Consistently, *in vitro* treatment of brown adipocytes with serotonin inhibited β3-adrenergic intracellular signaling, which was associated with lower *Ucp1* expression and reduced oxygen consumption. These data showed that the elevated peripheral serotonin due to maternal HFD contributes to the attenuation β3-adrenergic signaling in HFD BAT.

Lastly, we tested metformin administration during lactation on BAT function of HFD offspring, and observed astonishing recovery of HFD BAT function. Supportively, the serotonin concentration was elevated in HFD BAT, which was recovered due to metformin treatment. Because metformin is known to enhance lipid oxidation^44^, these data suggest that serotonin secretion is coupled with lipid metabolism in adipose tissue^25^. Of note, further studies are needed to explore the inhibitory effect of metformin on peripheral serotonin level in maternal HFD offspring, in addition to other likely mechanisms for the recovery actions of metformin on BAT thermogenic function of maternal HFD offspring.

In summary, to our best knowledge, for the first time, we observed that adaptive thermogenesis in HFD BAT was impaired at weaning, despite elevated *Ucp1* expression in BAT before cold exposure. Importantly, such BAT impairment due to HFD during lactation has lasting effects on BAT function in offspring, partially explaining the developmental programming of maternal HFD on offspring obesity and metabolic dysfunction. Lastly, the lactation stage represents an amiable time window for intervention to ensure offspring metabolic health.

## Methods

### Mice

Animal studies were conducted in AAALAC-approved facilities and according to protocols approved by the Institutional Animal Use and Care Committee (IAUCC) of Washington State University. To obtain offspring, 10 to12 week-old female C57/BL mice were mated. Success in mating was confirmed by the presence of vaginal plug and mice were continually maintained on chow diet until delivery. On the day of birth, litter sizes were balanced to 6 pups and then dams were randomly assigned to either control diet (Con; 10% energy from fat, D12450, Research Diets, New Brunswick, NJ) or high-fat diet (HFD; 60% energy from fat, D12492, Research Diets) during lactation. Animals were maintained under 12 h-light/12 h-dark cycles and with *ad libitum* access to water. The pups were weaned on chow diet on postnatal day 21 (PND21). For metformin treatment, newborn pups were administered daily with 200 mg/kg body weight metformin (Sigma, St. Louis, MO) dissolved in sterilized saline or saline only from PND2 to PND21^44^. To avoid confounding sexual effect, only male offspring were used in this study.

### Cell culture and treatment

Stromal vascular cells were isolated from BAT of one month old mice as previously described^22^. The brown adipogenesis was induced using an induction medium containing 15% fetal bovine serum (FBS), 5 μg/ml insulin, 1 μM dexamethasone, 0.5 mM isobutyl-1-methylxanthine, 125 nM indomethacin and 1 nM T3 for 2 days and subsequently maintained in a differentiation medium containing 5 μg/ml insulin and 1 nM T3.

Brown adipocytes were treated with serotonin as previously described^26^. Briefly, cells were treated with serotonin for 30 min (100 μM; Sigma) followed by isoproterenol (1 nM; Sigma) for 30 min or 4 h, respectively, and collected for western blotting or gene expression analyses. Coupled and uncoupled respiration rates of brown adipocytes were measured by addition of vehicle (1:1000) or 1 μM oligomycin (Sigma) dissolved with DMSO^45^, respectively. The O_2_ level was analyzed using an Orion Dissolved Oxygen platform (Thermo Scientific, Waltham, MA) as previously described^22^.

### Insulin and triglyceride assays

Blood triglyceride level was analyzed with triglyceride colorimetric assay kit from Cayman (Cat. 10010303; Ann Arbor, MI). Insulin level was analyzed with Mouse Ultrasensitive Insulin ELISA Kit (Cat. 80-INSMSU-E10; ALPCO Diagnostics, Salem, NH).

### Serotonin assay

BAT serotonin was extracted from frozen tissue samples using HOCL_4_. Homogenized samples were centrifuged at 12,000 × g for 5 min, and supernatants were carefully collected and neutralized using KOH. Neutralized supernatants were centrifuged at 12,000 × g for 10 min to remove any remaining precipitates. To isolate serotonin from serum, the same volume of serum of Con and HFD offspring was used. Proteins were precipitated by addition of cold acetonitrile, and precipitated proteins were removed by centrifugation as previously described^24^. Serotonin level was analyzed using HPLC as previously described^46^.

### Glucose and insulin tolerance test (GTT and ITT)

Mice were subjected to GTT (at 14-week of age) and ITT (at 15-week of age) as previously described^32^. Briefly, mice were fasted for 6 h before testing and, then, administered (i.p.) with 2 g/kg D-glucose or 0.75 U/kg insulin for GTT and ITT, respectively. Blood samples were collected from the tail veil at 0, 15, 30, 60 and 120 min post injection and glucose concentrations were measured by glucose oxidase method using a glucose meter (Bayer Contour, Tarrytown, NY, USA).

### Quantitative real-time PCR (qRT-PCR)

Total RNAs were extracted using TRIzol (Life technologies, Grand Island, NY) and cleaned with Zymo Research RNA clean & concentrator kit (Zymo research, Irvine, CA). cDNA was synthesized using an iScript™ cDNA Synthesis Kit (Bio-Rad, Hercules, CA) according to manufacturer’s instructions. qRT-PCR reactions were performed with SYBR green master mix (Bio-Rad, Hercules, CA) on a Bio-Rad real-time PCR unit. 18S rRNA was used as a reference gene. All primers are listed in Table 1.

### Western blotting

Analyses were conducted as previously described^32^. Antibodies including phospho-HSL (#4126), phospho-PKA substrate (#9624), and β-tubulin (#3873) were purchased from Cell Signaling Technology (Danvers, MA). Antibodies against UCP1 (SC-6529), HSL (SC-25843) and PPARα (SC-9000) were purchased from Santa Cruz Biotechnology (Santa Cruz, CA). IRDye 800CW goat anti-rabbit (Cat. 926-32211) and IRDye 680 goat anti-mouse (Cat. 926-68070) secondary antibodies were purchased from LI-COR Biosciences (Lincoln, NE). The target proteins were detected by using an Odyssey infrared imaging system (Li-cor Biosciences, Lincoln, NE) and bands were quantified using Software Image Studio Lite (LI-COR Biosciences, Version 4).

### Chromatin immunoprecipitation (ChIP)-PCR assay

Briefly, tissue samples were homogenized in liquid nitrogen, fixed in 1% formaldehyde solution, and then sonicated. The supernatant was precleaned with ChIP-grade protein G (Cell Signaling Technology) and then incubated with antibody against PPARα (SC-9000; Santa Cruz, CA), or normal rabbit IgG (#2729; Cell Signaling Technology, Danvers, MA). The antibody-chromatin complex was precipitated with protein G and further treated with RNaseA and proteinase K. DNA was purified with ChIP DNA clean & concentrator kit (Zymo Research, Irvine, CA) and used as templates for PCR using the primers listed in Table 1. Target enrichment was calculated after normalization to input^31^.

### Hematoxylin & Eosin (H&E) and Immunohistochemical staining

Fat tissues were fixed and sectioned into 5 μm thickness. H&E and immunohistochemical staining were conducted as previously described^46^. The ABC kit (Vector Laboratory Inc., Burlingame, CA) was used for antigen detection.

### Tissue oxygen (O_2_) consumption measurement

Tissue O_2_ consumption was determined as previously reported^47^. Approximately 10 mg of BAT tissue was minced with a fine scissors in a buffer comprised of 2% BSA, 1.1 mM sodium pyruvate, and 25 mM glucose in PBS. O_2_ consumption was measured using an Orion Dissolved Oxygen platform (Thermo Scientific, Waltham, MA) for approximately 20 min. O_2_ consumption was normalized to the BAT tissue weight.

### Oxygen consumption *in vivo*

Oxygen consumption of mice was measured in a Comprehensive Lab Animal Monitoring System (CLAMS, Columbus Instruments, OH, USA). Measurements were taken every 30 seconds for 24 h (12 h-light/12 h-dark cycle). Mice could access *ad libitum* to water and one pellet of diet to avoid fasting stress.

### Rectal temperature measurement

To monitor the rectal temperature, an animal temperature controller with a rectal probe for mice was used (Physitemp, Clifton, NJ). Temperature was measured every 2 h during the cold exposure.

### Surface interscapular BAT temperature

Surface interscapular BAT temperature was measured with an infrared camera (E6, FLiR Systems) as previously described^48^. Briefly, mice were i.p. injected with sterilized saline or 0.033 nmol/g of β3-adrenergic agonist CL-316, 243 (Santa Cruz Biotech). The images were acquired 20 min post injection. Then, mice were immediately euthanized for BAT collection for biochemical analysis.

### Statistical analysis

Data were presented as mean ± S.E.M. Results were analyzed using Student’s *t*-test or ANOVA where appropriate, with GraphPad Prism software (GraphPad Prism, version 6.0). *P* ≤ 0.05 was considered as significant difference.

## Additional Information

**How to cite this article**: Liang, X. *et al*. Maternal high-fat diet during lactation impairs thermogenic function of brown adipose tissue in offspring mice. *Sci. Rep.* **6**, 34345; doi: 10.1038/srep34345 (2016).

## Figures and Tables

**Table 1:** Sequences of primer used for real-time PCR and ChIP-PCR.

Gene	Forward (5′-3′)	Reverse (5′-3′)
*18s*	GTAACCCGTTGAACCCCATT	CCATCCAATCGGTAGTAGCG
*Cidea*	ATCACAACTGGCCTGGTTACG	TACTACCCGGTGTCCATTTCT
*Cox7α*	CAGCGTCATGGTCAGTCTGT	AGAAAACCGTGTGGCAGAGA
*Elovl3*	TCCGCGTTCTCATGTAGGTCT	GGACCTGATCCAACCCTATGA
*Fas*	TCTGCGATGAAGAGCATGGTTTAGAG	GTTTGTATTGCTGGTTGCTGTGCAC
*Lpl*	TCATCAACTGGATGGAGGAGGAGTT	AAGTTAGGCCCAGCTGGATCCA
*Pgc1a*	CCATACACAACCGCAGTCGC	GTGGGAGGAGTTAGGCCTGC
*Prdm16*	CAGCACGGTGAAGCCATTC	GCGTGCATCCGCTTGTG
*Pparα*	GGATGTCACACAATGCAATTCGC	GTTGAAGTTCTTCAGGTAGGCTTCGT
*Ucp1*	ACTGCCACACCTCCAGTCATT	CTTTGCCTCACTCAGGATTGG
*Ucp1 ChIP*	AAGAGAAGAACACGGACACTAGGTAAGT	TGGGAAGTGAAAGAAAGTTTGCC
